# Egg characteristics vary longitudinally in Arctic shorebirds

**DOI:** 10.1016/j.isci.2023.106928

**Published:** 2023-05-19

**Authors:** Jin Liu, Ziwen Chai, Hui Wang, Anton Ivanov, Vojtěch Kubelka, Robert Freckleton, Zhengwang Zhang, Tamás Székely

**Affiliations:** 1 Key Laboratory for Biodiversity Science and Ecological Engineering, College of Life Sciences, Beijing Normal University, Beijing 100875, China; 2 Institute of Ecology, College of Urban and Environmental Sciences, and Key Laboratory for Earth Surface Processes of the Ministry of Education, Peking University, Beijing 100871, China; 3 Timiryazev State Biological Museum, Malaya Grusinskaya, 15, Moscow 123242, Russia; 4 All-Russian Research Institute for Environmental Protection (ARRIEP), 36 km MKAD, Moscow 117628, Russia; 5 Department of Zoology and Centre for Polar Ecology, Faculty of Science, University of South Bohemia, Branišovská 1760, České Budejovice 37005, Czech Republic; 6 Ecology and Evolutionary Biology, School of Biosciences, University of Sheffield, Alfred Denny Building, Western Bank, Sheffield S10 2TN, UK; 7 Department of Evolutionary Zoology and Human Biology, Faculty of Science, University of Debrecen, Egyetem tér 1, Debrecen, Hungary; 8 Milner Centre for Evolution, University of Bath, Claverton Down, Bath BA2 7AY, UK

**Keywords:** Evolutionary biology, Ornithology, Zoology

## Abstract

Arctic environments are changing rapidly and if we are to understand the resilience of species to future changes, we need to investigate alterations in their life histories. Egg size and egg shape are key life-history traits, reflecting parental investment as well as influencing future reproductive success. Here we focus on egg characteristics in two Arctic shorebirds, the Dunlin (*Calidris alpina*) and the Temminck’s stint (*Calidris temminckii*). Using egg photos that encompass their full breeding ranges, we show that egg characteristics exhibit significant longitudinal variations, and the variation in the monogamous species (Dunlin) is significantly greater than the polygamous species (Temminck’s stint). Our finding is consistent with the recent “disperse-to-mate” hypothesis which asserts that polygamous species disperse further to find mates than monogamous species, and by doing so they create panmictic populations. Taken together, Arctic shorebirds offer excellent opportunities to understand evolutionary patterns in life history traits.

## Highlights

• Egg characteristics exhibit longitudinal variations in two Arctic shorebird species
• Egg characteristics vary more in monogamous Dunlins than polygamous Temminck’s stints
• The geographic variations in eggs are consistent with the “disperse-to-mate” hypothesis

## Graphical abstract

**Figure:**
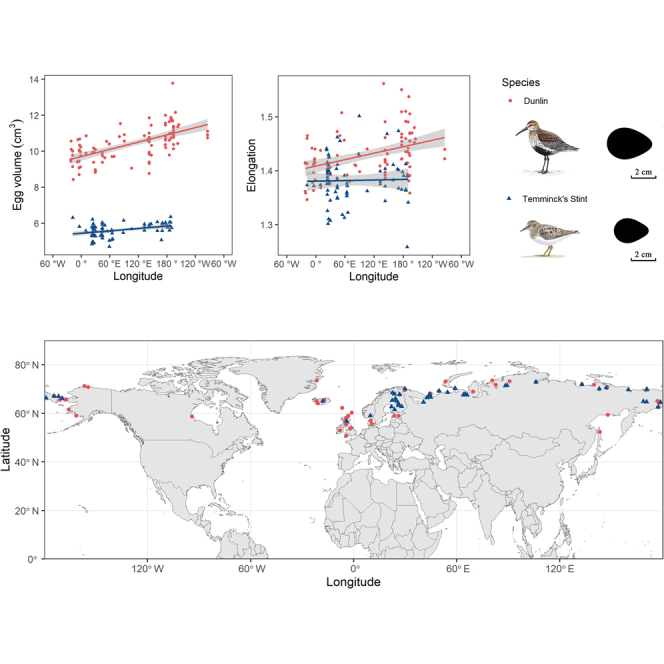

## Introduction

The female gamete (ovum or egg) comes in different shapes and sizes in birds: For example, egg shape ranges from spherical, oval to pointed shapes, and egg mass ranges from 0.1g up to nearly 2 kg in extant birds.^1^^,^^2^ The variety of egg shape and size has long fascinated scientists, consequently, there are excellent historic data, specimens and information on eggs of the vast majority of bird species. Intra- (and inter-) specific variations in eggs are increasingly exploited by evolutionary studies that seek to understand the origins and adaptative mechanisms shaping variations in egg characteristics.^3^^,^^4^ For instance, such studies suggest that egg shape can be an adaptation for incubation efficiency,^5^^,^^6^ the type of breeding site and incubation posture,^7^^,^^8^ and it might be related to high-powered flight,^4^ climate, and nest structure.^9^ Furthermore, egg size has been considered an important life-history trait, which usually reflects parental investment in reproduction.^10^^,^^11^^,^^12^^,^^13^ Egg size is also associated with offspring fitness, because large eggs are usually more hatched at a more advanced stage of development and possess greater nutrient reserves.^14^

Egg characteristics are usually heritable,^15^^,^^16^^,^^17^^,^^18^^,^^19^^,^^20^ and they can also be influenced by the condition of the female and/or the environment. For example, egg size can be affected by female body size^21^ and food quality,^17^^,^^22^ whereas egg shape can be influenced by soil calcium through the process of eggshells formation.^23^^,^^24^ The intraspecific variations of egg characteristics can be maintained by reduced of gene flow between neighboring populations or they can be the result of adaptation to the local environment. Investigating the variations both within a species and within populations is crucial to understand the potential ecological, evolutionary and physiological causes of phenotypic differences in egg characteristics.

Mating system may also impact intraspecific variation in egg characteristic through gene flow. Previous studies of temperate and tropical shorebirds proposed an intriguing association between mating systems and gene flow by arguing that males in polygamous species should disperse widely during the breeding season to find new mates, and these movements are expected to increase gene flow within and between populations (the disperse-to-mate hypothesis).^25^^,^^26^ Although the hypothesis was supported by genetic data in plovers *Charadrius* spp. and by the different rates of speciation that showing slower diversification in polygamous shorebirds than in monogamous ones,^25^^,^^26^ the relevance of the hypotheses across a broader range of taxa has remained contested. Recently, satellite-tracking presented an independent line of evidence by showing that the polygamous male Pectoral sandpipers (*Calidris melanotos*) covers 1000s of kilometers during the breeding season in the high Arctic while searching for new mates.^27^ To follow up these lines of investigations, we hypothesized that a monogamous Arctic shorebird exhibits larger geographic variations because of reduced gene flow between distant breeding grounds than a polygamous shorebird.

**Figure 1:**
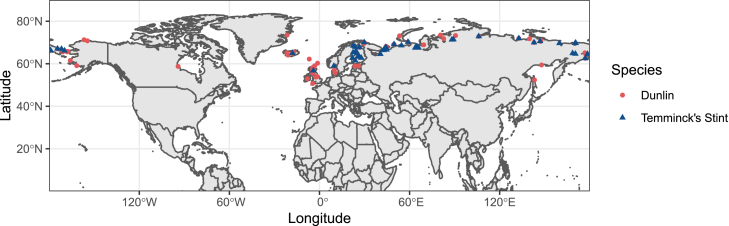
Geographic locations of Dunlin and Temminck’s stint clutches included in the study (n = 96 and 74 clutches, respectively)

Here we focus on two common Arctic shorebirds (Dunlin *Calidris alpina* and Temminck’s stint *Calidris temminckii*) to explore intraspecific variation in their egg characteristics. The Arctic tundra offers an excellent wild laboratory for speciation and diversification, because unlike in most temperate and tropical landscapes that are fragmented by geographic barriers such as high mountains or deserts, the Arctic tundra is a largely continuous circumpolar habitat within which animals can move relatively freely. It encompasses a variety of habitats and climate conditions along longitude, and such gradients are expected to contribute to intraspecific variations of life-history traits, because of the adaptation to local environmental conditions.^28^^,^^29^^,^^30^^,^^31^ The objectives in the present study are to investigate geographic variations in egg characteristics in both Dunlin and Temminck’s stint and to test whether the “disperse-to-mate” hypothesis is applicable to phenotypic variations in egg characteristics. Dunlins are socially and genetically monogamous whereas Temminck’s stints have a variable mating system whereby both the male and female attain multiple mates during a single breeding season.^32^^,^^33^^,^^34^ Dunlin and Temminck’s stint are widely distributed in the Arctic region (Figure 1), with simple open nests on the ground and a constant clutch size of four eggs.^35^^,^^36^ The extensive genetic differentiations in Dunlins and the reduced genetic variations in Temminck’s stint found by previous studies provide an excellent opportunity to test the “disperse-to-mate” hypothesis.^37^^,^^38^^,^^39^^,^^40^^,^^41^

We took egg photographs at various museum collections to quantify geographic variations in egg characteristics. We first report that egg characteristics exhibit strong longitudinal patterns that might be influenced by climatic gradient along longitude and intraspecific variations in female size. In particular, eggs in the eastern populations are larger in both species than the western populations, eggs are more pointed and more elongated and eggshells are heavier in the eastern populations for Dunlin. Second, we find egg characteristics vary more significantly with longitude in the monogamous species (Dunlin) than in the polygamous one (Temminck’s stint), suggesting that dispersal emerging from the different mating systems might influence the extent of geographic variation in egg characteristics.

## Results

### Intraspecific variation in egg characteristics

**Figure 2:**
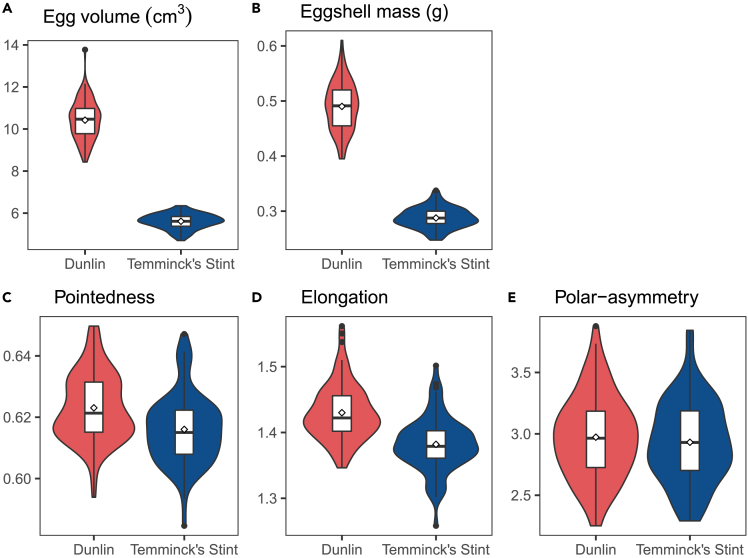
Intraspecific variation of egg size and egg shape characteristics in Dunlin (DL) and Temminck’s stint (TS) (n = 96 and 74 clutches respectively for the analyses of egg volume, pointedness, elongation and polar-asymmetry; whereas n = 88 and 69 clutches for the analyses of eggshell mass) (A) Egg volume; (B) eggshell mass; (C) pointedness; (D) elongation; (E) polar-asymmetry. The violin plots and boxplots show the distribution, the median, first and third quartile and 1.5× interquartile range of the egg characteristics, whereas the diamonds provide the mean values.

Eggs of Dunlin were larger ($V_{DL}=10.422,V_{Ts}=5.613,t=46.65,p<0.001)$, more pointed $\left(P_{DL}=0.623,P_{TS}=0.616,t=4.04,p<0.001\right)$ and more elongated ($E_{DL}=1.430,E_{TS}=1.382,t=7.43,p<0.001)$ with heavier eggshells ($S_{DL}=0.490,S_{TS}=0.287,t=39.04,p<0.001)$ than eggs of Temminck’s stint, whereas polar-asymmetry was not statistically different between the two species ($R_{DL}=2.973,R_{TS}=2.931,t=0.80,p=0.425)$. The variance in egg characteristics were not statistically different between the two species, except egg volume $\left(F_{\left(95,73\right)}=6.78,p<0.001\right)$ and eggshell mass $\left(F_{\left(87,68\right)}=5.62,p<0.001\right)$, because Dunlin eggs were more variable than Temminck’s stint eggs (Figure 2, Table 1).

**Table 1:** Intraspecific variation of egg shape and egg size in two Arctic shorebirds

Egg characteristics	Dunlin			Temminck’s stint			Mean comparison		Variance comparison	
	mean	SD	CV	mean	SD	CV	t	p	F	p
Egg volume (cm^3^)	**10.422**	0.93	8.88	**5.613**	0.36	6.33	46.65	**<0.001**	F_(95,73)_ = 6.78	**<0.001**
Eggshell mass (g)	**0.490**	0.04	8.96	**0.287**	0.02	6.44	39.04	**<0.001**	F_(87,68)_ = 5.62	**<0.001**
Pointedness	**0.623**	0.01	1.78	**0.616**	0.01	1.85	4.04	**<0.001**	F_(95,73)_ = 0.95	0.794
Elongation	**1.430**	0.04	2.95	**1.382**	0.04	3.01	7.43	**<0.001**	F_(95,73)_ = 1.02	0.919
Polar-asymmetry	**2.973**	0.34	11.34	2.931	0.33	11.39	0.80	0.425	F_(95,73)_ = 1.02	0.936

Notes: “mean” refers to the mean value, “SD” refers to the Standard deviation, “CV” refers to the Coefficient of variation, “t” refers to t value, “F” refers to F value, “p” refers to p value. The statistically significant values (p < 0.05) are bolded. See also Table S3.

### Geographic variation in egg characteristics

**Figure 3:**
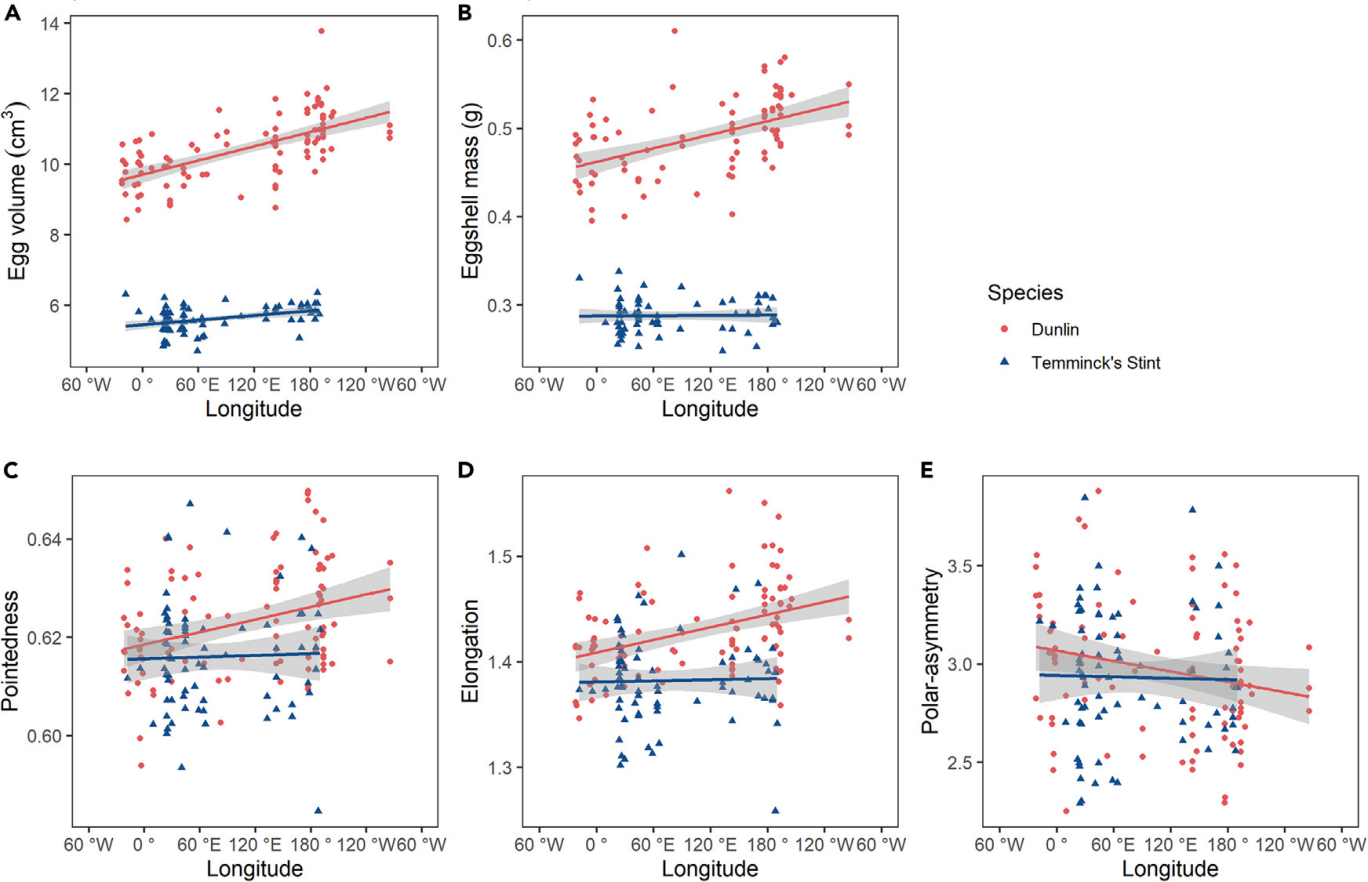
Egg characteristics in relation to longitude (A) Egg volume; (B) eggshell mass; (C) pointedness; (D) elongation; (E) polar-asymmetry. Lines are generated from linear regressions, and the gray area shows 95% confidence intervals.

Egg characteristics exhibited strong geographic patterns (Figure 3, Table S1): in Dunlin, egg volume ($slope=7.3\times 10^{-3},t=7.45,p<0.001$), eggshell mass ($slope=3.15\times 10^{-4},t=5.81,p<0.001$), pointedness $\left(slope=3.48\times 10^{-5},t=2.46,p=0.016\right)$ and elongation $\left(slope=1.79\times 10^{-4},t=3.48,p\;<\;0.001\right)$ increased with longitude, whereas polar-asymmetry decreased with longitude $\left(slope=-8.4\times 10^{-4},t=-1.85,p=0.067\right)$. In Temminck’s stint, geographic variation was non-significant except egg volume that increased with longitude $\left(slope=2.5\times 10^{-3},t=3.18,p=0.002\right)$. Egg characteristics did not change between years (Table S1).

The aforementioned patterns were confirmed when we directly compared eggs between Dunlins and Temminck’s stints (Figure 3, Table 2), because geographic variation was significantly larger in the monogamous species, the Dunlin than in Temminck’s stint in egg volume ($slope_{DL}=6.70\times 10^{-3},slope_{TS}=2.19\times 10^{-3},F_{\left(1,166\right)}=11.88,p<0.001$), eggshell mass ($slope_{DL}=2.56\times 10^{-4},slope_{TS}=5.00\times 10^{-6},F_{\left(1,153\right)}=12.09,p<0.001$), and elongation ($slope_{DL}=2.00\times 10^{-4},slope_{TS}=1.77\times 10^{-5},F_{\left(1,166\right)}=4.19,p=0.04$). The steeper geographic gradients of the monogamous species were apparent in all five egg characteristics (Figure 3, Table 2).

**Table 2:** Geographic variation in egg characteristics in two Arctic shorebirds: Dunlin (DL) and Temminck’s stint (TS)

Egg characteristics	Species	Estimate intercepts of Species				Estimate slopes of Species × Longitude (con)				F test of Species × Longitude (con)		adj R^2^
		Estimate	SE	t	p	Estimate	SE	t	p	F	p	
Egg volume (n = 96, 74)	DL	**9.30**	0.131	70.90	<0.001	**6.70×10**^**−**^**^3^**	7.00 × 10^−4^	9.60	**<0.001**	F_(1,166)_ = 11.88	**<0.001**	0.95
	TS	**5.32**	0.211	−18.90	<0.001	**2.19×10**^**−**^**^3^**	1.31 × 10^−3^	−3.45	**<0.001**			
Eggshell mass (n = 88,69)	DL	**0.45**	7.45 × 10^−3^	59.95	**<0.001**	**2.56×10**^**−**^**^4^**	3.93 × 10^−5^	6.53	**<0.001**	F_(1,153)_ = 12.09	**<0.001**	0.91
	TS	**0.29**	1.19 × 10^−2^	−13.40	**<0.001**	**5.00×10**^**−**^**^6^**	7.23 × 10^−5^	−3.48	**<0.001**			
Pointedness (n = 96, 74)	DL	**0.616**	2.44 × 10^−3^	252.62	**<0.001**	**4.25×10**^**−**^**^5^**	1.30 × 10^−5^	3.28	**0.001**	F_(1,166)_ = 2.28	0.13	0.13
	TS	0.615	3.92 × 10^−3^	−0.18	0.856	5.78 × 10^−6^	2.43 × 10^−5^	−1.51	0.133			
Elongation (n = 96, 74)	DL	**1.40**	8.95 × 10^−3^	156.08	**<0.001**	**2.00×10**^**−**^**^4^**	4.76 × 10^−5^	4.21	**<0.001**	F_(1,166)_ = 4.19	**0.04**	0.31
	TS	1.38	1.44 × 10^−2^	−1.18	0.238	**1.77×10**^**−**^**^5^**	8.92 × 10^−5^	−2.05	**0.04**			
Polar-asymmetry (n = 96, 74)	DL	**3.12**	0.07	42.01	**<0.001**	**−8.8×10**^**−**^**^4^**	3.95 × 10^−4^	−2.23	**0.027**	F_(1,166)_ = 1.05	0.31	0.02
	TS	3.12	0.12	−1.44	0.151	−7.6 × 10^−4^	7.41 × 10^−4^	1.03	0.306			

Notes: The form of the regression is *Y* ∼ Species × Longitude (con) (*Y*: clutch means of egg volume, eggshell mass, pointedness, elongation, and polar-asymmetry). “n” refers to the number of clutches for DL and TS respectively, “con” represents the converted longitude, “adj R^2^” represents the adjusted R^2^. “estimate” refers to the estimate intercepts or the estimate slopes, “SE” refers to the standard error of the estimate, “t” refers to the t value, “p” refers to the p value, “F” refers to the F value. The model is the parsimonious version of the model in Table S2. The statistically significant values (p < 0.05) are bolded. See also Table S4.

### Associations among egg characteristics

Larger eggs had heavier eggshell in both species ($\rho_{DL}=0.78,p<0.001;\;\rho_{TS}=0.59,p<0.001)$, and larger eggs tended to be more elongated in Dunlin ($\rho_{DL}=0.26,p<0.001;\;\rho_{TS}=-0.001,p=0.985)$. Eggs with heavier eggshells were more elongated ($\rho_{DL}=0.19,p<0.001;\;\rho_{TS}=0.16,p<0.001)$ and more pointed ($\rho_{DL}=0.09,p=0.09;\;\rho_{TS}=0.15,p=0.012)$ in both species. Larger and more elongated eggs were less asymmetric, and such association was more apparent in Dunlin ($\rho_{DL}=-0.12,p=0.028;\;\rho_{TS}=-0.04,p=0.515)$. In addition, more pointed eggs were more asymmetric in both species as expected ($\rho_{DL}=0.40,p<0.001,\rho_{TS}=0.32,p<0.001)$ (Table 3).

**Table 3:** Associations between volume, shell mass, and shape parameters of individual eggs using Pearson correlation

Temminck’s stint	Dunlin									
	Egg volume (n = 360)		Eggshell mass (n = 343)		Elongation (n = 360)		Pointedness (n = 360)		Polar-asymmetry (n = 360)	
	ρ	p	ρ	p	ρ	p	ρ	p	ρ	p
Egg volume (n = 285)	/		**0.78**	**<0.001**	**0.26**	**<0.001**	**0.10**	**0.048**	**−0.12**	**0.028**
Eggshell mass (n = 270)	**0.59**	**<0.001**	/		**0.19**	**<0.001**	0.09	0.086	−0.08	0.128
Elongation (n = 285)	−0.001	0.985	**0.16**	**0.009**	/		**0.22**	**<0.001**	**−0.27**	**<0.001**
Pointedness (n = 285)	−0.02	0.696	**0.15**	**0.012**	**0.41**	**<0.001**	/		**0.40**	**<0.001**
Polar-asymmetry (n = 285)	−0.04	0.515	0.06	0.360	−0.09	0.114	**0.32**	**<0.001**	/	

Notes: “n” refers to the number of eggs, “ρ” refers to the correlation coefficient, “p” refers to the p value. The statistically significant values (p < 0.05) are bolded. See also Table S5.

## Discussion

Our study yielded three main findings. First, we found that the monogamous species, Dunlin, exhibited larger geographic variation in egg morphology than the polygamous species – Temminck’s stint. Second, we found longitudinal trends of egg size in both species: eggs in the east are larger. We also found longitudinal trends of egg shape in Dunlin: eggs in the east are larger, more pointed and more elongated and eggshells are heavier, whereas eggs in the west are on the opposite. Third, the allometric associations within species are consistent with previous inter-specific analyses, except for the association between elongation with polar-asymmetry and the association between egg volume with polar-asymmetry.

Egg variations were consistent with our expectation in that the monogamous species exhibited greater geographic variation than the polygamous species. Although the pattern was significant in three out of five egg characteristics, the trend was in the same direction in all five traits. These two species often breed side by side and their life histories are very similar, apart from their mating system. The “disperse-to-mate” hypothesis suggests that polygamous species promote intense sexual selection with mating dispersal, hence leading to widespread gene flow across the breeding range.^26^^,^^42^ Our finding is consistent with the hypothesis, and also consistent with systematic studies of these two species: there are nine subspecies in Dunlin,^40^^,^^41^^,^^43^ whereas Temminck’s stints are monotypic with no clear population genetic structure across the whole breeding range.^38^^,^^39^ Our results suggest a stronger local adaptation of egg characteristics in Dunlin than in Temminck’s stint.

We propose four nonexclusive explanations for the more extensive geographic differentiations among Dunlin populations than in Temminck’s stint. (1) The Dunlin may be more residential and less migratory than Temminck’s stints at breeding sites^44^^,^^45^^,^^46^^,^^47^^,^^48^ – a behavior independent from their mating system – and this may generate stronger geographic differentiation in Dunlin. However, we are not aware of any evidence that would point in this direction. (2) Because egg shape and size can be influenced by clutch size, larger clutch size is often related to smaller egg size and less spherical shape,^5^^,^^6^^,^^49^ larger geographic variations in clutch size in Dunlins could potentially produce the patterns we reported here. However, as far as we can tell, both species produce four eggs in vast majority of clutches (n = 96 and 74 clutches for DL and TS respectively, $F_{\left(1,168\right)}=0.24,p=0.624$), so the clutch size variation may not account for the different within-species variation in the two species. (3) Past glaciations may have influenced the two species differently. For instance, Dunlins could have retreated to several different refugia during the maximum extent of ice cover, and successive fragmentation of populations could be because of these different refugia.^40^^,^^41^ However, it seems that Temminck’s stints used fewer refugia and (or) emerged from these limited number of refugia more recently than Dunlins, hence having lower genetic diversity and lower geographic variation in egg characteristics.^38^^,^^39^ (4) Egg characteristics may be more limited in Temminck’s stint than in Dunlin because of stronger heritability, although we are not aware data currently that would allow to evaluate this potential explanation. To sum up this section, we argue that the first two explanations are unlikely, although the latter two explanations –with the “disperse-to-mate” hypothesis^26^ – remain viable.

The longitudinal pattern of egg characteristics might be influenced by climatic factors in the circumpolar area or it might be affected by female body size. Eggs closer to the western side of Eurasia are smaller, less elongated and less pointed, whereas eggs closer to the eastern side are the opposite. Similar longitudinal patterns of egg characteristics have also been found in several shorebirds, including Northern lapwing (*Vanellus vanellus*), Red-necked phalarope (*Phalaropus lobatus*), Common ringed plover (*Charadrius hiaticula*) and Ruddy turnstone (*Arenaria interpres*).^50^^,^^51^ Because oceanic climate also varies along longitudinal gradients in the Arctic area, the local precipitation and temperature likely to impact on food availability via phenology and affect reproductive effort for females during incubation and hence on egg characteristics.^52^^,^^53^^,^^54^ In addition, the geographic patterns of eggs in Dunlin and Temminck’s stint are consistent with the trend in female body size along longitude,^55^ where female body size is smaller in the west but larger in the east, and such variation is greater in Dunlin than in Temminck’s stint (Figure S7 and Table S6). In contrast to latitudinal patterns in life histories that have been widely tested,^56^^,^^57^^,^^58^ longitudinal variations have remained understudied. We call for further examinations on other species and using further life-history traits to validate general patterns, and also to find out the mechanisms that influencing reproductive outputs of Arctic shorebirds.

Although climate change is rapidly influencing Arctic ecosystems,^59^^,^^60^^,^^61^^,^^62^ we did not find temporal variations in egg characteristics in Dunlins nor Temminck’s stints. Perhaps more detailed temporal analyses – by restricting the samples to those areas which have multiple samples from over a large time span – could challenge the findings we report here.

Finally, the negative associations between elongation and polar-asymmetry, and the ones between egg volume and polar-asymmetry somehow contradict previous findings in seabirds. This could support Birkhead et al.^8^ suggestion that the associations are an adaptation for incubating on cliffs exhibited by various seabirds, whereas Dunlins and Temminck’s stints nest on the ground, so the incubation period might not influence polar-asymmetry in these two shorebirds. However, because larger eggs and more elongated eggs are more symmetrical, for a four-egg clutch, such associations might be beneficial to maximize the thermal transfer from females, such that these associations could be another adaptive way to contribute to incubation efficiency.^5^^,^^6^

In conclusion, we show that egg characteristics exhibit significant longitudinal pattern in Dunlin and Temminck’s stint, and mating system may modulate the degree of geographic variations of egg characteristics. Our study raises intriguing patterns and provides an Arctic perspective on the evolution of egg characteristics. Although we only use here data from two species, there is a need to investigate egg characteristics of other Arctic species by using multiple independent phylogenetic events to test the longitudinal patterns and the “disperse-to-mate” hypothesis across a broader range of species. Ultimately, these studies will help understanding how egg morphology adapt to the changing environment, and further exploring the mechanisms of speciation and diversification in the rapidly changing Arctic environment.

### Limitations of study

We only use data from two species to investigate the geographic variation; there is a need to investigate egg characteristics of other Arctic species by using multiple independent phylogenetic events to test the longitudinal patterns and the “disperse-to-mate” hypothesis across a broader range of species.

## STAR★Methods

### Key resources table

**Table:** 

REAGENT or RESOURCE	SOURCE	IDENTIFIER
**Deposited data**		

Egg characteristics	This paper	https://doi.org/10.17632/nr5r9zzytb.1
R code for the statistical analysis	This paper	https://doi.org/10.17632/nr5r9zzytb.1

**Software and algorithms**		

R	R Core Team^1^	https://www.R-project.org/
GPS coordinates converter	NA	https://www.gps-coordinates.net/gps-coordinates-converter
Adobe DXO Viewpoint 3 Version 3.1.6	Adobe	https://www.dxo.com/zh-cn/dxo-viewpoint/
Adobe Photoshop CS6 Version13.0	Adobe	https://www.adobe.com/cn/products/photoshop.html

**Other**		

Egg photographing protocol	Biggins et al.^2^	https://doi.org/10.1002/ece3.4412
Egg shape analysis methodology	Biggins et al.^3^	https://doi.org/10.5061/dryad.8kv2b20
Illustration of Dunlin in the graphical abstract	Birds of the World, by Francesc Jutglar	https://doi.org/10.2173/bow.dunlin.01
Illustration of Temminck’s stint in the graphical abstract	Birds of the World, by Francesc Jutglar	https://doi.org/10.2173/bow.temsti.01

### Resource availability

#### Lead contact

Further information and requests for resources should be directed to and will be fulfilled by the lead contact, Zhengwang Zhang (zzw@bnu.edu.cn).

#### Materials availability

This study did not generate new unique reagents.

### Experimental model and study participant details

361 eggs from 96 clutches of Dunlin (*C. alpina*) and 285 eggs from 74 clutches of Temminck’s stint (*C. temminckii*) were used in our analyses. Collected year of clutches ranges from 1874 to 2016 in Dunlin (DL), and ranges from 1855 to 2014 in Temminck’s stint (TS); collected date of clutches ranges from the 126^th^ day of the year to the 199^th^ day of the year in DL, and ranges from the 158^th^ day of the year to the 216^th^ day of the year in TS; clutch size ranges from one to four in DL and one to seven in TS, but is mostly four in both species; collected latitude ranges from 50.71°N to 73.50°N in DL and ranges from 56.79°N to 72.89°N in TS; collected longitude ranges from 174.50°W to 177.49°E in DL and ranges from 179.12°W to 178.53°E in TS (Figure S4). No other subjects are included in this study.

### Method details

#### Data collection

We measured and photographed eggs from three museum collections: the Natural History Museum at Tring (BNHM, UK), the Western Foundation of Vertebrate Zoology (WFVZ, USA) and the Zoological Museum of Moscow University (ZMMU, Russia). We studied all available eggs of each clutch, excluding eggs with broken shells or that were fragile to hold.

Using museum labels, we recorded the following information about each clutch: species, clutch size, number of eggs that are available for measurement, collection date (including year, month and day), collection locality and name of collectors (Figure S4). For collection localities that lacked geographic coordinates, the collection location was used to estimate latitude and longitude via a GPS coordinates converter (www.gps-coordinates.net/gps-coordinates-converter) in decimal degrees format.^61^ We did not include clutches for which collection locality or collection date was ambiguous, and excluded clutches away from the normal breeding range of the species to avoid recording errors. A total of 361 eggs (from 96 clutches) of Dunlin and 285 eggs (from 74 clutches) of Temminck’s stint were used in the analyses (Figure 1).

#### Egg measurement and photographing

We measured the maximum length (in mm) and maximum breadth (in mm) of each egg using a digital caliper. We measured the eggshell mass (in gram) of each egg by using an electronic balance. Because most eggs were blown to clear using a small hole, we also measured the diameter (in mm) for round holes or measured the length and the width for rectangle holes by using a straight ruler, to consider hole size in the analyses of eggshell mass.

We used a standard protocol designed by Biggins et al.^63^ to photograph eggs. Eggs were held by an egg holder, and a set square was located to ensure that each egg was positioned with its long axis parallel to both the lightpad and the camera lens. Eggs were placed on the center of the MiniSun A4 LED Modern Ultra-slim Art Lightpad, using a spirit level to ensure each egg was at a horizontal level in all directions (Figure S1).

Photographs were taken using a Canon EOS 7D Mark II DSLR Camera with a Canon EF100mm f/2.8L IS USM Macro lens, attached to a Manfrotto 128RC tripod head, mounted on a Manfrotto 055XPROB tripod stand. The horizontal column axis was set so that the camera lens was facing directly onto the lightpad. A scale was set on the camera, to make sure that the camera was level and parallel to the lightpad.

The distance between the camera and the lightpad was 40 cm when the photographing was conducted in BNHM and ZMMU, and was 37 cm when the photographing was conducted in WFVZ. To get the silhouette photograph (the egg outline) of each egg (Figure S2), we used the following manual settings in all three museums: Focal length 100 mm, F-stop: f/20, Exposure time: Auto, ISO speed: 200. Photos were subsequently corrected for potential lens distortion using Adobe DXO Viewpoint 3 Version 3.1.6, and photos with poor contrast were edited to adjust the contrast by Adobe Photoshop CS6 Version13.0 before any shape analysis.

#### Shape analysis

The shape analysis followed the methodology developed by Biggins et al*.*^63^ We used the packages Preston.R and Indices.R^64^ to process the photographs in R program. We inputted the maximum length and maximum breadth of each egg we measured, together with the silhouette photograph of each egg. We derived four variables that characterise the size and shape of eggs: (a) pointedness, the length from the point where the egg is widest to the more distant end divided by the overall length, which is P/L (Figure S3); (b) elongation, the ratio of the length to the width at the widest point, which is L/D (Figure S3); (c) polar-asymmetry, the ratio of the diameter of the largest circle that can fit within the egg outline and touch the egg at its blunt pole to the diameter of the largest circle within the egg outline and touching the more pointed pole, which is $R_{B}/R_{P}$ (Figure S3); (d) egg volume.^63^

### Quantification and statistical analysis

We focus on five parameters: egg volume (*V* cm^3^), eggshell mass (*S* g), together with three variables to indicate egg shape: pointedness (*P*), elongation (*E*) and polar-asymmetry (*R*). Eggshell mass measured in museums might be lighter than their real mass, because blow holes in eggshells used to make specimens may reduce the mass of the shell. Therefore, before any statistical analyses we first tested the effects of the number and the size of blow holes on eggshell mass, and did not find any significant association (Figure S5 and Table S7). So we used the measured eggshell mass in the analyses.

To test whether geographical location contributes to the intraspecific variances of egg characteristics, we fitted linear models for each egg characteristic for two species separately, with latitude (denoting it as *m*) and longitude (denoting it as *n*) were the predictors. Egg characteristics were estimated at clutch-level by calculating the mean value of each clutch for each trait. As the Arctic has been experiencing dramatic climate change, affecting animals’ reproduction and survival,^60^^,^^61^^,^^62^ we considered year (denoting it as *y*) and date (denoting it as *t*) in the models to correct the temporal variations. The museum collections included three somehow abnormal clutches that had five, six and seven eggs in one clutch (Figure S4), we run the key models by including clutch size in our models (denoting it as *c*).

All predictors were continuous variables. Longitude and latitude were expressed in decimal degrees format. Both species have circumpolar distribution with a gap in the North American arctic (Figure S6),^38^^,^^39^^,^^40^^,^^41^ so we use 60°W as the zero-reference point for modelling longitude in our statistical models. Year refers to the collection year of the clutch recorded on museum labels; date refers to the collection date of the clutch and it is used as Julian date in the models; clutch size refers to the number of eggs recorded on the museum labels. Since we had *a priori* predictions (see the introduction), we kept all predictors in the model to assess their statistical significance. The regression equations were in the following form:

(Equation 1) $$Y=\alpha_{1}+\beta_{11}\left(m\right)+\beta_{12}\left(n\right)+\beta_{13}\left(y\right)+\beta_{14}\left(t\right)+\beta_{15}\left(c\right)+\varepsilon_{1}$$

where *Y* refers to clutch means of *V, S, P, E,* and *R*.

To compare the extent of longitudinal variation in egg characteristics between Dunlin and Temminck’s stint (Tables 1 and S1), we fitted linear models for each response variable (see Table S2). The most parsimonious models were in the following form:

(Equation 2) $$Y=\alpha_{2}+\beta_{21}\left(n\right)+\beta_{22}\left(a\right)+\beta_{23}\left(a\times n\right)+\varepsilon_{2}$$

where *a* refers to species, and *Y* refers to clutch means of *V, S, P, E,* and *R*.

To test the allometric associations between egg characteristics, we used Pearson correlation coefficients (*ρ*) between two egg characteristics at a time using individual egg-level data. We centered each egg characteristic for each species, so that the slopes between models are comparable. All models were performed in R program,^65^ results with p<0.05 are considered as statistically significant.

## Data Availability

• The data of egg characteristics from two of the three museums has been deposited at Mendeley, and is publicly available as of the date of publication. The rest data will be deposited at Mendeley once the museum approves. The DOI is listed in the key resources table. • All original code has been deposited at Mendeley, and is publicly available as of the date of publication. The DOI is listed in the key resources table. • Any additional information required to reanalyze the data reported in this paper is available from the lead contact upon request.
