# Supplementary material for: Egg characteristics vary longitudinally in Arctic shorebirds
Source: iScience. 2023 May 19;26(6):106928. doi: 10.1016/j.isci.2023.106928 (PMC10250164; doi:10.1016/j.isci.2023.106928)
Supplement: Document S1. Figures S1–S7 and Tables S1–S7 [file mmc1.pdf]

iScience, Volume *26*

Supplemental information

Egg characteristics vary longitudinally in Arctic shorebirds

Jin Liu, Ziwen Chai, Hui Wang, Anton Ivanov, Vojtěch Kubelka, Robert Freckleton, Zhengwang Zhang, and Tamás Székely

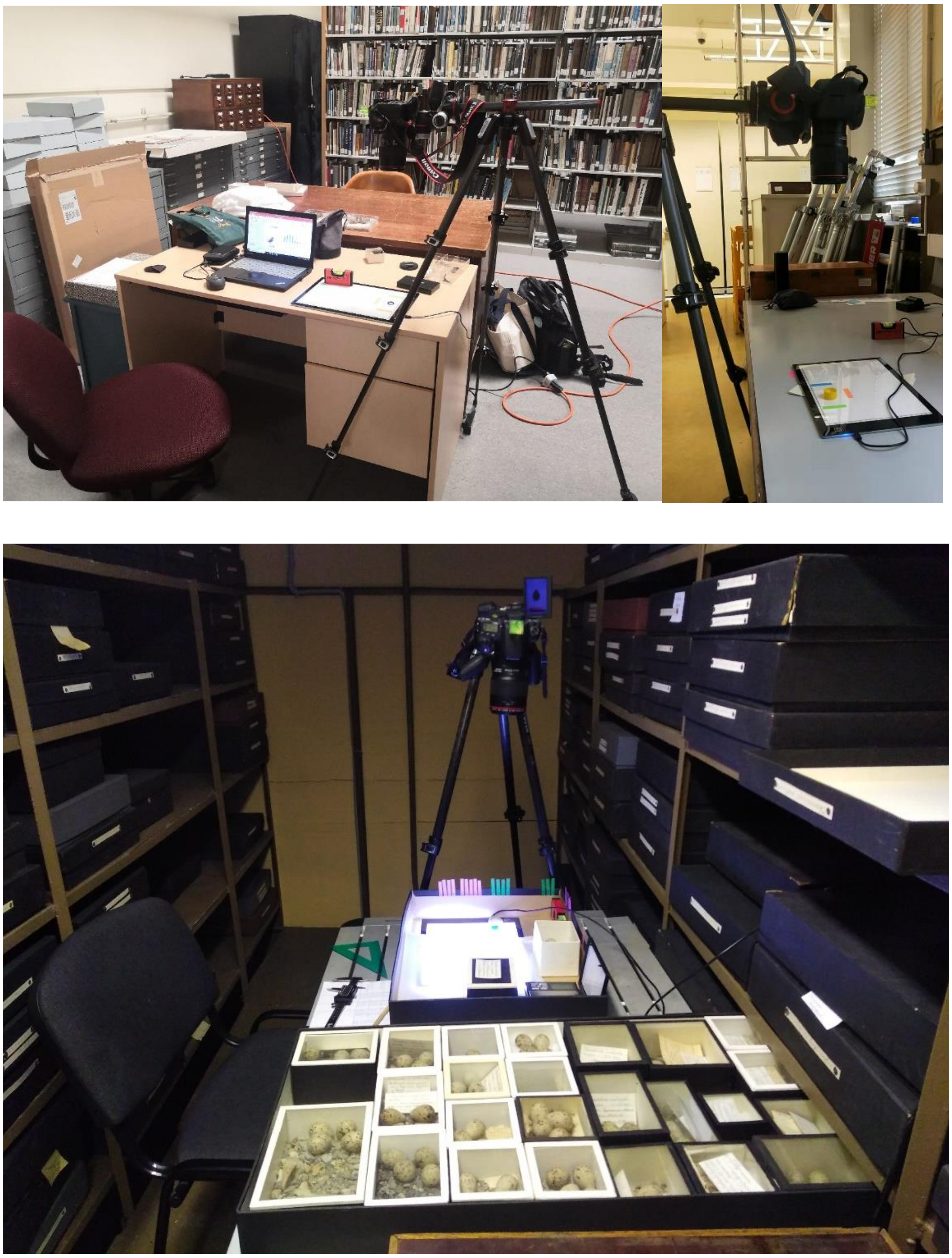

Figure S1. The set of devices when photographing eggs in the WFVZ (top left), BNHM (top right) and ZMMU (bottom) museum, related to STAR Methods.

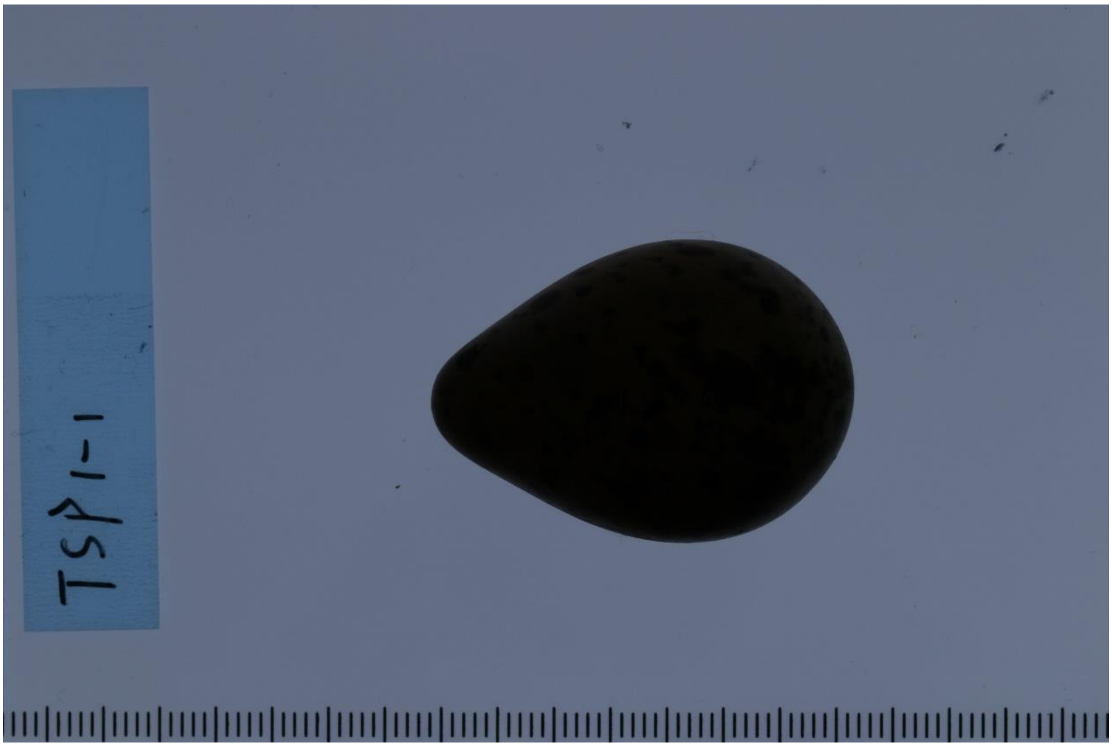

Figure S2. An example of the silhouette photograph of an egg taken from the museum, related to STAR Methods.

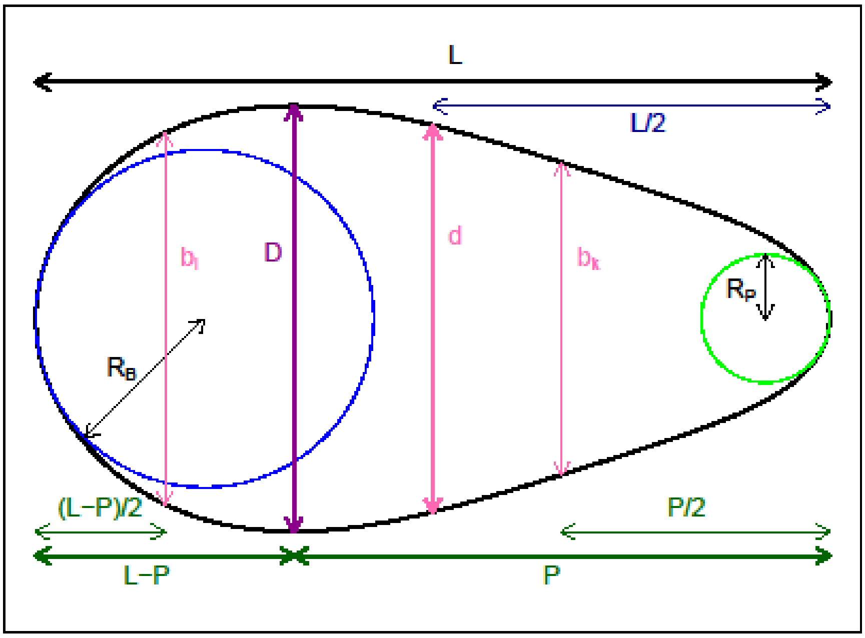

Figure S3. Parameters of egg formulae (copyright [S1]), related to STAR Methods.

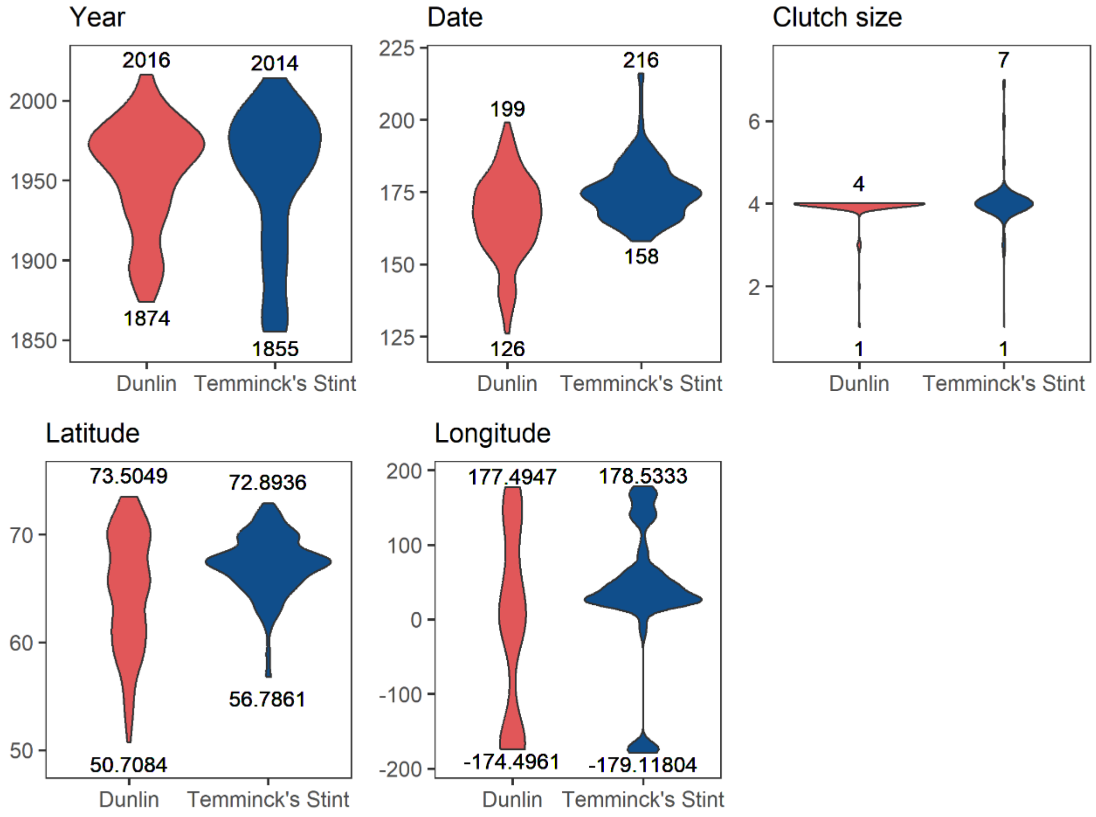

Figure S4. The distribution of predictors, related to STAR Methods.

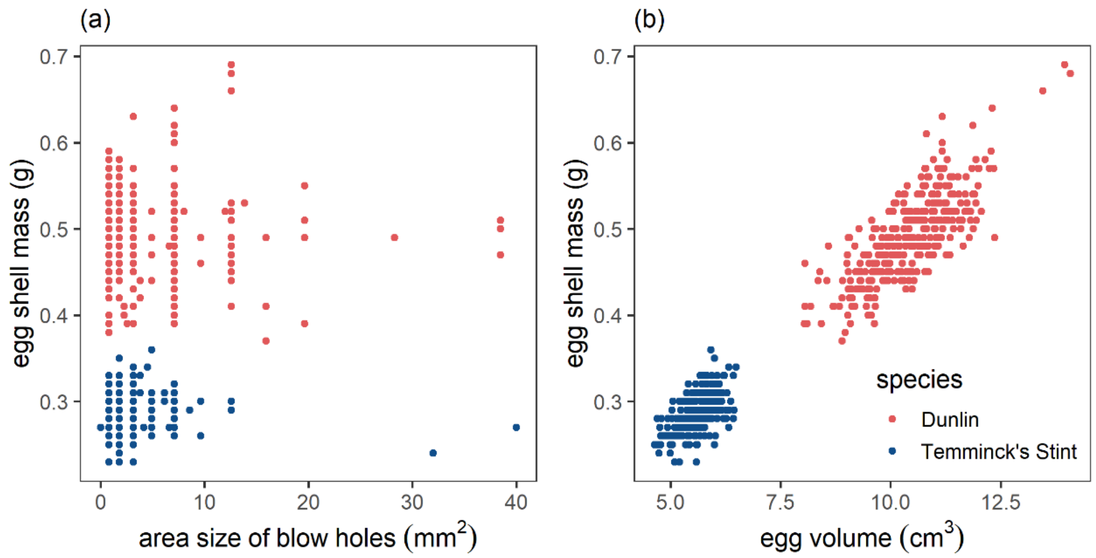

Figure S5. The relationship between the area size of blow holes, egg volume and eggshell mass, related to STAR Methods. (a) The relationship between the area size of blow holes and eggshell mass. (b) The relationship between egg volume and eggshell mass. Colour represents species.

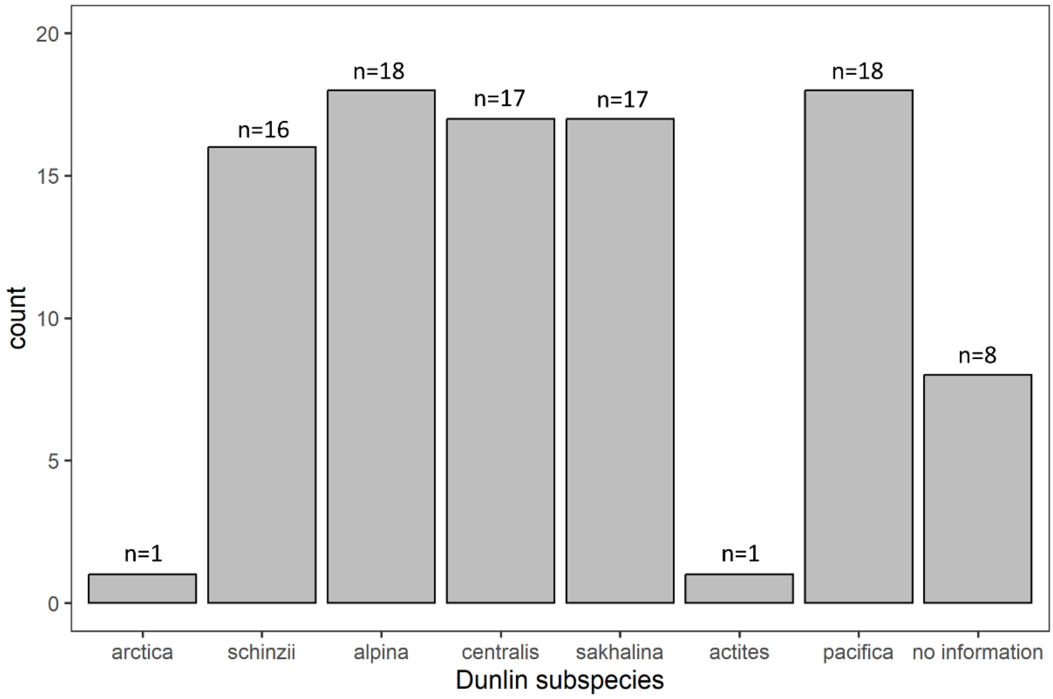

Figure S6. Sample size of Dunlin subspecies, related to STAR Methods. Subspecies are ordered following their breeding distribution from the west to the east. “No information” represents the clutches without subspecies information.

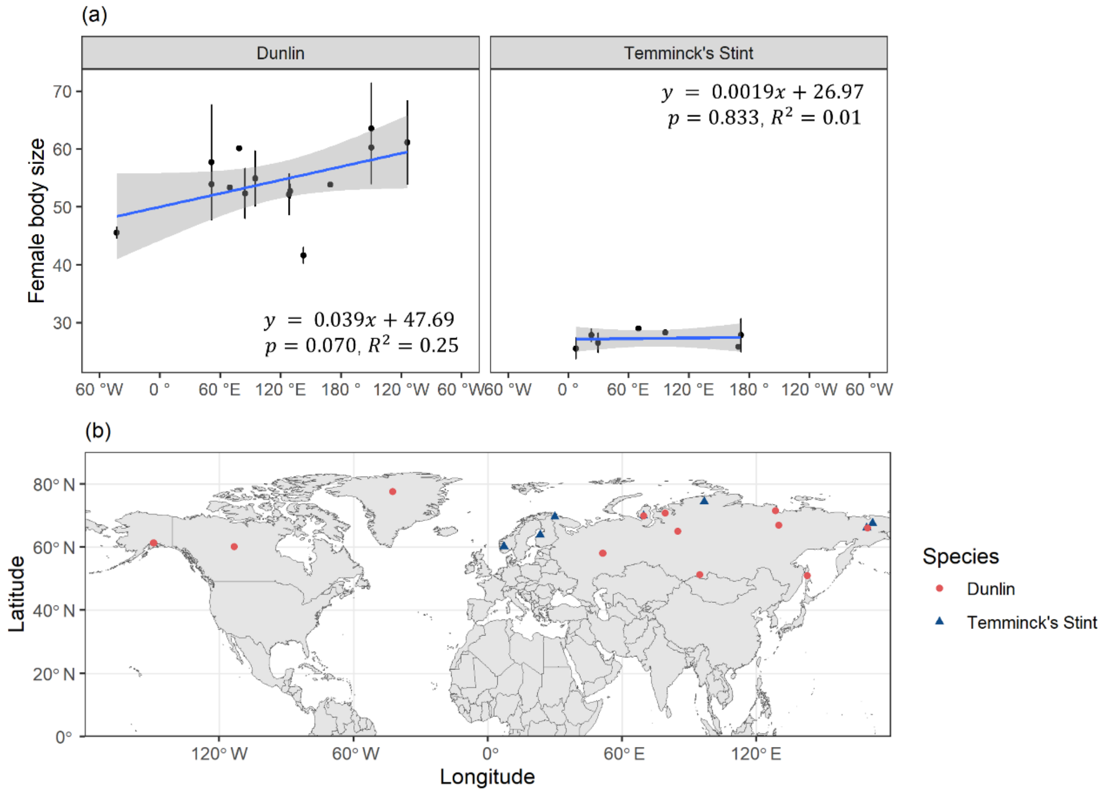

Figure S7. The longitudinal trends of female body size for Dunlin and Temminck's Stint, related to Figure 3. (a) The longitudinal trends of female body size for two species. Points are the mean values and bars are the standard deviations, both of them are reported by literature. Lines are predicted by linear regressions, the model, the p-value and the adjusted R^2 are reported. (b) The geographic distribution of female body size sample. Female body size collected from published literature, and the detailed information can be found in Table S6.

Table S1. Intraspecific variations of egg characteristics for Dunlin and Temminck's Stint, related to STAR Methods.

	Latitude	Longitude (con)	Year	Date	Clutch size	adj R^2
**Dunlin**						
Egg volume (n = 96)	-6.62×10^{-3} (SE = 0.02, t = -0.43, p =0.669)	7.3×10^{-3} (SE = 9.78×10^{-3}, t=7.45, **$p < 0.001$**)	-4.8×10^{-3} (SE = 2.50×10^{-3}, t = -1.92, p = 0.058)	4.71×10^{-3} (SE = 6.48×10^{-3}, t = 0.73, p = 470)	-0.09 (SE = 0.14, t = -0.68, p = 0.496)	0.39
Eggshell mass (n = 88)	-5.27×10^{-3} (SE = 8.42×10^{-4}, t = -0.63, p = 0.533)	3.15×10^{-4} (SE = 5.42×10^{-5}, t = 5.81, **$p < 0.001$**)	-2.8×10^{-4} (SE = 1.46×10^{-4}, t = -1.93, t = 0.058)	-1.4×10^{-4} (SE = 3.56×10^{-4}, t = -0.39, p = 0.694)	-1.3×10^{-2} (SE = 7.18, t = -1.88, p = 0.064)	0.27
Pointedness (n = 96)	1.74×10^{-4} (SE = 2.24×10^{-4}, t = 0.78, p = 0.439)	3.48×10^{-5} (SE = 1.42×10^{-5}, t = 2.46, **$p = 0.016$**)	5.62×10^{-5} (SE = 3.63×10^{-5}, t = 1.55, p = 0.124)	-6.48×10^{-5} (SE = 9.40×10^{-5}, t = -0.69, p = 0.493)	-7.09×10^{-4} (SE = 2.00×10^{-3}, t = -0.35, p = 0.725)	0.10

Elongation (n = 96)	3.47×10^{-4} (SE = 8.12 $\times10^{-4}$, t = 0.43, p = 0.671)	1.79×10^{-4} (SE = 5.15×10^{-5}, t = 3.48, **p < 0.001**)	1.18×10^{-4} (SE = 1.32×10^{-4}, t = 0.90, p = 0.372)	-9.92×10^{-5} (SE = 3.41×10^{-4}, t = -0.291, p = 0.772)	-1.38×10^{-2} (SE = 7.27×10^{-3}, t = -1.89, p = 0.062)	0.18
Polar-asymmetry (n = 96)	5.48×10^{-3} (SE = 7.15 $\times10^{-3}$, t = 0.77, p =0.446)	-8.4×10^{-4} (SE = 4.53×10^{-4}, t = -1.85, p = 0.067)	7.92×10^{-6} (SE = 1.16×10^{-3}, t = 0.06, t = -0.25, p = 0.995)	-1.67×10^{-3} (SE = 3.00×10^{-3}, t = -0.56, p = 0.580)	-0.02 (SE = 0.06, t = -0.25, p = 0.802)	0.01
**Temminck's Stint**						
Egg volume (n = 74)	6.93×10^{-3} (SE = 0.01, t = -0.47, p = 0.635)	2.5×10^{-3} (SE = 7.89×10^{-4}, t = 3.18, **p = 0.002**)	2.14×10^{-4} (SE = 1.10×10^{-3}, t = -0.19, p = 0.847)	-9.04×10^{-4} (SE = 3.94×10^{-3}, t= -0.23, p = 0.820)	0.09 (SE = 0.06, t = 1.52, p = 0.133)	0.13
Eggshell mass (n = 69)	-8.48×10^{-4} (SE = 9.49 $\times10^{-4}$, t = -0.89, p = 0.375)	-1.83×10^{-5} (SE = 4.54×10^{-5}, t = -0.40, p = 0.687)	9.17×10^{-5} (SE = 6.57×10^{-5}, t = 1.40, p = 0.168)	-1.18×10^{-4} (SE = 2.65×10^{-4}, t = -0.45, p = 0.656)	7.28×10^{-4} (SE = 3.42×10^{-3}, t = 0.21, p = 0.832)	-0.03

Pointedness (n = 74)	2.01×10^{-4} (SE = 5.07×10^{-4}, t = 0.40, p = 0.693)	4.37×10^{-6} (SE = 2.75×10^{-5}, t = 0.16, p =0.874)	1.99×10^{-6} (SE = 3.84×10^{-5}, t = 0.05, p = 0.959)	8.53×10^{-5} (SE = 1.38×10^{-4}, t = 0.62, p = 0.537)	3.23×10^{-3} (SE = 1.98×10^{-3}, t = 1.63, p = 0.108)	-0.03
Elongation (n = 74)	-5.68×10^{-5} (SE = 1.86×10^{-3}, t = -0.03, p = 0.976)	2.47×10^{-5} (SE = 1.01×10^{-4}, t = 0.25, p = 0.807)	2.28×10^{-5} (SE = 1.41×10^{-4}, t = 0.16, p = 0.872)	-5.24×10^{-4} (SE = 5.04×10^{-4}, t = -1.04, p = 0.302)	4.61×10^{-3} (SE = 7.26×10^{-3}, t = 0.64, p = 0.527)	-0.03
Polar-asymmetry (n = 74)	0.02 (SE = 0.01, t = 1.22, p = 0.23)	-3.83×10^{-4} (SE = 8.11×10^{-4}, t = -0.47, p = 0.638)	1.60×10^{-4} (SE = 1.13×10^{-3}, t = 0.14, p = 0.888)	-3.53×10^{-3} (SE = 4.05×10^{-3}, t = -0.87, p = 0.387)	5.25×10^{-3} (SE = 0.06, t = 0.09, p = 0.929)	-0.04

Notes: The form of the regression is *Y* ~ Latitude + Longitude (con) + Year + Date + Clutch size (*Y*: clutch means of egg volume, eggshell mass, pointedness, elongation, and polar-asymmetry). Numbers are estimated slopes, “n” refers to the number of clutches, “con” represents the converted longitude, “adj R^2” represents the adjusted R^2. The statistically significant values ($p<0.05$) are bolded.

Table S2. The full models of the geographic variations analysis, related to STAR Methods.

	Intercept for Species	Slope for Latitude	Slope for Date	Slope for Year	Slope for Clutch size	Slope for Longitude (con)	Species× Longitude (con)	adj R^2
Egg volume (n = 96, 74)	DL: 13.13 (SE = 2.68, t = 4.90, **p<0.001**); TS:9.06 (SE = 0.23, t = -17.71, **p<0.001**) $F_{1,162}$ = 2482.27, **p < 0.001**	-3.53×10^{-3} (SE = 0.011), $F_{1,162}$ = 0.36, p = 0.548)	1.21×10^{-3} (SE = $4.13\times10^{-3)}$, $F_{1,162}$ = 0.18, p = 0.669	-2.03×10^{-3} (SE = 1.37×10^{-3}), $F_{1,162}$ = 5.66, **p = 0.019**	0.028 (SE = 0.073), $F_{1,162}$ = 0.36, p = 0.550	DL: 7.02×10^{-3} (SE = 7.62 $\times10^{-4}$, t = 9.21, **p<0.001**); TS: -3.89×10^{-3} (SE = 1.38×10^{-3}, t=-2.82, **p=0.005**)	$F_{1,162}$ = 7.93, **p = 0.005**	0.94
Eggshell mass (n = 88, 69)	DL: 0.65 (SE = 0.15, t = 4.28, **p<0.001**); TS: 0.49 (SE=0.01, t = -12.27, **p<0.001**), $F_{1,149}$ =	-4.41×10^{-4} (SE = 6.30×10^{-4}), $F_{1,149}$ = 2.47, p = 0.118	-2.62×10^{-4} (SE=2.41×10^{-4}). $F_{1,149}$ = 0.23, p =	-5.93×10^{-5} (SE = 7.90×10^{-5}), $F_{1,151}$ = 2.20, p =	-4.87×10^{-3} (SE = 3.95×10^{-3}), $F_{1,149}$ = 0.94, p =	DL: 2.82×10^{-4} (SE = 4.27 $\times10^{-5}$, t = 6.61, **p < 0.001**); TS: 3.46×10^{-5} (SE = 7.52	$F_{1,149}$ = 10.79, **p = 0.001**	0.91

	1485.75, **$p < 0.001$**		0.634	0.140	0.335	$\times 10^{-5}$, $t = -3.29$, **$p = 0.001$**),		
Pointedness (n = 96, 74)	DL: 0.54 (SE = 0.05, $t = 10.92$, **$p < 0.001$**); TS: 0.54 (SE = 4.28×10^{-3}, $t = -0.048$, $p = 0.962$), $F_{1,162} = 12.15$, **$p < 0.001$**	1.57×10^{-4} (SE = 2.06×10^{-4}), $F_{1,166} = 0.81$, $p = 0.369$	-7.92×10^{-7} (SE = 7.67×10^{-5}), $F_{1,165} = 0.0001$, $p = 0.993$	3.00×10^{-5} (SE = 2.54×10^{-5}), $F_{1,164} = 0.41$, $p = 0.528$	1.25×10^{-3} (SE = 1.36×10^{-3}), $F_{1,163} = 1.09$, $p = 0.298$	DL: 3.61×10^{-5} (SE = 1.42×10^{-5}, $t = 2.55$, **$p = 0.012$**); TS: -7.87×10^{-6}, SE = 2.56×10^{-5}, $t = -1.72$, $p = 0.088$), $F_{1,168} = 13.50$, **$p < 0.001$**	$F_{1,162} = 2.95$, $p = 0.088$	0.12
Elongation (n = 96, 74)	DL: 1.20 (SE = 0.18, $t = 6.56$, **$p < 0.001$**); TS: 1.19 (SE = 0.02, $t = -0.62$, **$p <0.001$**), $F_{1,167} = 46.80$, **$p < 0.001$**	4.85×10^{-4} (SE = 7.58×10^{-4}), $F_{1,166} = 0.21$, $p = 0.645$	-2.39×10^{-4} (SE = 2.82×10^{-4}), $F_{1,165} = 0.08$, $p = 0.776$	1.10×10^{-4} (SE = 9.36×10^{-5}), $F_{1,164} = 0.37$, $p = 0.541$	-2.42×10^{-3} (SE = 5.01×10^{-3}), $F_{1,163} = 0.09$, $p = 0.762$	DL: 1.91×10^{-4} (SE = 5.21×10^{-5}, $t = 3.66$, **$p < 0.001$**), TS: -3.51×10^{-6} (SE = 5.01×10^{-3}, $t = -0.48$, **$p = 0.018$)**, $F_{1,168} =$	$F_{1,162} = 5.73$, **$p = 0.02$**	0.30

						26.24, **$p < 0.001$**		
Polar-asymmetry (n = 96, 74)	DL: 2.54 (SE = 1.52, t = 1.67, p = 0.097); TS: 2.40 (SE = 0.13, t = -1.06, p = 0.29), $F_{1,167}$ = 1.39, p = 0.241	8.30×10^{-3} (SE = 6.29×10^{-3}), $F_{1,166}$ = 1.078, p = 0.301	-2.51×10^{-3} (SE = 2.34×10^{-3}), $F_{1,165}$ = 1.56, p = 0.213	2.31×10^{-4} (SE = 7.77×10^{-4}), $F_{1,164}$ = 0.26, p = 0.613	1.06×10^{-3} (SE = 0.04), $F_{1,163}$ = 0.001, p = 0.978	DL: -8.51×10^{-4} (SE = 4.32×10^{-4}, t = -1.97, p = 0.051); TS: 5.59×10^{-4} (SE = 7.82×10^{-4}, t = 0.71, p = 0.476), $F_{1,168}$ = 3.16, p = 0.077	$F_{1,162}$ = 0.51, p = 0.476	0.02

Notes: The form of the regression is *Y* ~ Latitude + Year + Date + Clutch size + Species × Longitude (*Y*: clutch means of egg volume, eggshell mass, pointedness, elongation, and polar-asymmetry). "n" refers to the number of clutches for DL and TS respectively, "con" represents the converted longitude, "adj R^2" represents the adjusted R^2. The model is the full model of Table 2. The statistically significant values ($p < 0.05$) are bolded.

Table S3. Intraspecific variation of egg shape and egg size in two Arctic shorebirds (*excluding the abnormal clutches with more than four eggs), related to Table 1.

	Dunlin	Temminck's Stint	Mean comparison	Variance comparison
Egg volume (cm^3)	**10.422** (sd = 0.93, cv = 8.88)	**5.608** (sd = 0.36, cv = 6.44)	t = 46.40, **p < 0.001**	$F_{(95,70)}$ = 6.56, **p <0.001**
Eggshell mass (g)	**0.490** (sd = 0.04, cv = 8.96)	**0.287** (sd = 0.02, cv = 6.41)	t = 39.06**, p < 0.001**	$F_{(87,65)}$ = 5.70, **p < 0.001**
Pointedness	**0.623** (sd = 0.01, cv = 1.78)	**0.615** (sd = 0.01, cv = 1.78)	t = 4.39**, p < 0.001**	$F_{(95,70)}$ = 1.02, p = 0.949
Elongation	**1.430** (sd = 0.04, cv = 2.95)	**1.381** (sd = 0.04, cv = 3.01)	t = 7.52**, p < 0.001**	$F_{(95,70)}$ = 1.03, p = 0.913
Polar-asymmetry	**2.973** (sd = 0.34, cv = 11.34)	2.936 (sd = 0.34, cv = 11.56)	T = 0.699, p = 0.486	$F_{(95,70)}$ = 0.99, p = 0.945

Notes: Three abnormal clutches of Temminck’s Stint were excluded, including one 5-eggs clutch, one 6-eggs clutch and one 7-eggs clutch.

Table S4. Geographic variations in egg characteristics in two Arctic shorebirds (excluding the abnormal clutches with more than four eggs), related to Table 2.

	Estimate intercepts of Species	Estimate slopes of Species×Longitude (con)	F test of Species×Longitude	adj R^2
Egg volume (n = 96, 71)	**DL: 9.30** (SE = 0.132, t = 70.34 **$p < 0.001$**)	**DL: 6.70×10^{-3}** (SE = 7.04×10^{-4},t = 9.53, **$p < 0.001$**)	$F_{1,163}$ = 11.34, **$p < 0.001$**	0.94
	**TS: 5.30** (SE = 0.215, t = -18.64, **$p < 0.001$**)	**TS: 2.25×10^{-3}** (SE=1.32×10^{-3}, t = -3.37, **$p < 0.001$**)		
Eggshell mass (n = 88,66)	**DL: 0.45** (SE = 7.49×10^{-3}, t = 59.63, **$p < 0.001$**)	**DL: 2.56×10^{-4}** (SE = 3.95×10^{-5}, t = 6.49, **$p < 0.001$**)	$F_{1,150}$ = 11.32, **$p < 0.001$**	0.91
	**TS: 0.29** (SE = 1.21×10^{-2}, t = -13.30, **$p < 0.001$**)	**TS: 1.07×10^{-5}** (SE = 7.30×10^{-5}, t = -3.37, **$p < 0.001$**)		
Pointedness (n = 96, 71)	**DL: 0.616** (SE=2.40×10^{-3}, t = 257.17, **$p < 0.001$**)	**DL: 4.25×10^{-5}** (SE = 1.27×10^{-5}, t = 3.34, **$p = 0.001$**)	$F_{1,163}$ = 1.85, p = 0.176	0.15
	TS: 0.614 (SE = 3.89×10^{-3}, t = -0.47, p = 0.638)	TS: 9.88×10^{-6} (SE = 2.40×10^{-5}, t = -1.36, p = 0.175)		
Elongation (n	**DL: 1.40**	**DL: 2.00×10^{-4}**	$F_{1,163}$ = 3.82, p = 0.052	0.32

= 96, 71)	(SE = 8.94×10^{-3}, t = 156.31, **p < 0.001**)<br>TS: 1.38<br>(SE = 1.45×10^{-2}, t = -1.33, p = 0.18)	(SE = 4.75×10^{-5}, t = 4.22, **p < 0.001**)<br>TS: 2.57×10^{-5}<br>(SE = 8.94×10^{-5}, t = -1.95, p = 0.052)		
Polar-asymmetry (n = 96, 71)	**DL: 3.12**<br>(SE = 0.07, t = 41.72, **p < 0.001**)<br>TS: 2.96<br>(SE = 0.12, t = -1.34, p = 0.182)	**DL: -8.8×10^{-4}**<br>(SE = 3.98×10^{-4}, t = -2.21, **p = 0.028**)<br>TS: -1.55×10^{-4}<br>(SE = 7.48×10^{-4}, t = 0.97, p = 0.334)	$F_{1,163} = 0.939$, p = 0.334	0.01

Notes: The form of the regression is *Y* ~ Species × Longitude(con) (*Y*: clutch means of egg volume, eggshell mass, pointedness, elongation, and polar-asymmetry). Numbers are estimated values, “n” refers to the number of clutches for DL and TS respectively, “con” represents the converted longitude, “adj R^2” represents the adjusted R^2. The model is the parsimonious version of the model in Table S2. The statistically significant values ($p < 0.05$) are bolded. Three abnormal clutches of Temminck’s Stint were excluded, including one 5-eggs clutch, one 6-eggs clutch and one 7-eggs clutch.

Table S5. Associations between volume, shell mass and shape parameters of individual eggs using Pearson correlation (excluding the abnormal clutches with more than four eggs), related to Table 3.

Dunlin / Temminck's Stint	Egg volume (n = 360)	Eggshell mass (n = 343)	Elongation (n = 360)	Pointedness (n = 360)	Polar-asymmetry (n = 360)
Egg volume (n = 267)	/	**0.78 (p < 0.001)**	**0.26 (p < 0.001)**	**0.10 (p = 0.048)**	**-0.12 (p = 0.028)**
Eggshell mass (n = 252)	**0.59 (p < 0.001)**	/	**0.19 (0 < 0.001)**	0.09 (p = 0.086)	-0.08 (p = 0.128)
Elongation (n = 267)	-0.02 (p = 0.689)	**0.14 (p = 0.029)**	/	**0.22 (p < 0.001)**	**-0.27 (p < 0.001)**
Pointedness (n = 267)	-0.06 (p = 0.313)	0.72 (p = 0.256)	**0.40 (p < 0.001)**	/	**0.40 (p < 0.001)**
Polar-asymmetry (n = 267)	-0.03 (p = 0.575)	-0.03 (p = 0.636)	-0.08 (p = 0.171)	**0.36 (p < 0.001)**	/

Notes: “n” refers to the number of eggs. Three abnormal clutches of Temminck’s Stint were excluded, including one 5-eggs clutch, one 6-eggs clutch and one 7-eggs clutch. The statistically significant values ($p < 0.05$) are bolded.

Table S6. The information of female body size collected from published literature, related to Figure 3.

Country	Location	n	Female, g M±SD (lim)	Source
**Temminck's stint** *Calidris temminckii*				
N Norway	Varanger	29	26.5 ± 1.7 (22.1-31.3)	Lislevand, T., Marthinsen, G., and Lifjeld, J.T. (2009). Sex differences in body size and body condition in breeding Temminck's Stints *Calidris temminckii*. J. Ornithol 150, 299-302. Lislevand, T. (2017). Part 12: Ageing and sexing the Temminck's Stint *Calidris temminckii*. Wader Study 124, 55–59.
S Norway	Hardangervidda	32	25.5 ± 1.8	Lislevand, T. (2017). Part 12: Ageing and sexing the Temminck's Stint *Calidris temminckii*. Wader Study 124, 55–59.
Finland	Kokkola (63°52'N. 23°05'E)	28	27.8 ± 1.19 (26.0-29.5)	Hildén, O. (1975). Breeding system of Temminck's stint *Calidris temminckii*. Ornis Fenn 52, 117-146.
Russia	Yamal	1	29.0	Ryabitsev, V.K. (2007). Temminck's stint *Calidris temminckii* in Yamal. Russ. J. Ornithol 16, 1191-1208.
Russia	Central and Eastern Taimyr	2	28.3 ± 0.4 (28.0-28.5)	Chupin, I.I. (2002). Birds of Central and Eastern Taimyr (ecology, population, zoogeography). Thesis for the degree of candidate of sciences, 369.
Russia	Yakutia	4	27.8 ±2.9	Kretchmar, A.V., Andreev, A.V., and Kondratyev, A.Ya. (1991). Birds of Northern plains (Nauka Published House).
Russia	Chukotka	20	25.8	Kondratiev, A.Ya. (1982). Biology of waders in the tundra of Northeast Asia, 191.
**Dunlin** *Calidris alpina*				
				Calidris alpina arctica
Greenland	Greenland	5	45.5 ± 1.0	Engelmoer, M., and Roselaar, C.S. (1998). Geographical Variation in Waders (Kluwer Academic Publishers).

				Calidris alpina alpina
Fennoscandia - Russia	Fennoscandia - Yamal	10	52.3 ± 4.3	Engelmoer, M., and Roselaar, C.S. (1998). Geographical Variation in Waders (Kluwer Academic Publishers).
Russia	Yamal Peninsula	3	53.3 (51.0-57.0)	Danilov, N.N., Ryzhanovsky, V.N., and Ryabitsev, V.K. (1984). Birds of Yamal 1, 332.
Russia	Gydan Peninsula	5	60.1* (50.3-74.7)	Zhukov, V.S. (2014). Evaluation of morphometric data of the dunlin *Calidris alpina* in the tundra zone of the West Siberian Plain. Russ. J. Ornithol 23, 957-972.
				Calidris alpina centralis
Russia	Taimyr. Medusa Bay	11	53.9 ± 3.3	Tulp, I., and H, Schekkerman. (2001). Studies on breeding shorebirds at Medusa Bay, Taimyr, in summer 2001. Wageningen. Alterra. Green World Research. Alterra-report 451, 110.
Russia	Central and Eastern Taimyr	3	57.7 ± 10.0 (50.0-69.0)	Chupin, I.I. (2002). Birds of Central and Eastern Taimyr (ecology, population, zoogeography). Thesis for the degree of candidate of sciences, 369.
Russia	Taimyr - Kolymsk	5	52.1 ± 3.6	Engelmoer, M., and Roselaar, C.S. (1998). Geographical Variation in Waders (Kluwer Academic Publishers).
Russia	Yakutia	2	52.7 ± 1.3	Kretchmar, A.V., Andreev, A.V., and Kondratyev, A.Ya. (1991). Birds of Northern plains (Nauka Published House).
				Calidris alpina sakhalina
Russia	NE Siberia	15	54.9 ± 4.8	Engelmoer, M., and Roselaar, C.S. (1998). Geographical Variation in Waders (Kluwer Academic Publishers).
Russia	Chukotka	45	53.8	Kondratiev, A.Ya. (1982). Biology of waders in the tundra of Northeast Asia, 191.
				Calidris alpina actites
Russia	Sakhalin Island	3	41.6 ± 1.4	Engelmoer, M., and Roselaar, C.S. (1998). Geographical Variation in Waders (Kluwer Academic Publishers).

				Calidris alpina pacifica
USA	W &S Alaska	23	60.2 ± 6.3	Engelmoer, M., and Roselaar, C.S. (1998). Geographical Variation in Waders (Kluwer Academic Publishers).
				Calidris alpina articola
USA	N Alaska	14	63.5 ± 7.9	Engelmoer, M., and Roselaar, C.S. (1998). Geographical Variation in Waders (Kluwer Academic Publishers).
Canada	N Canada	13	61.1 ± 7.3	Engelmoer, M., and Roselaar, C.S. (1998). Geographical Variation in Waders (Kluwer Academic Publishers).

Notes: "n" represents the number of individuals, "M" represents mean values, "SD" represents standard deviations, "*" represents that there was one female with an egg in the oviduct.

Table S7. The relation between the size of blow holes and eggshell mass, related to STAR Methods.

	Dunlin		Temminck's Stint	
Egg volume (cm^3)	$F_{1,342} = 16.168$	$p < 0.001$	$F_{1,269} = 9.498$	$p < 0.001$
The area of blow holes (mm^2)	$F_{1,341} = 0.866$	$p = 0.388$	$F_{1,268} = 0.921$	$p = 0.358$

Notes: The area size of each blow hole is calculated by using the diameter (the shape of the hole is round) or the length and width (the shape of the hole is a rectangle). The linear mixed effects models were conducted for each species respectively, models are in the form: eggshell mass ~ egg volume + hole area + (1| clutch ID).

Supplemental references

S1. Biggins, J.D., Thompson, J.E., and Birkhead, T.R. (2018). Accurately quantifying the shape of birds' eggs. Ecol. Evol *8*, 9728-9738. 10.1002/ece3.4412.
